# Antiseizure medications and their differing effects on cardiovascular risk^☆^

**DOI:** 10.1016/j.ebr.2025.100746

**Published:** 2025-02-01

**Authors:** Aleena Abbasi, Bassil Abbasi, Scott Mintzer, Carla LoPinto-Khoury

**Affiliations:** a Department of Neurology, Temple University, Philadelphia, PA, United States; b Department of Neurology, Thomas Jefferson University, Philadelphia, PA, United States

**Keywords:** Antiseizure medications (ASMs), Cardiovascular risk, Epilepsy management, enzyme-inducing ASMs (EIASMs), non-enzyme-inducing ASMs (NEIASMs), Lipid profile, C-reactive protein (CRP), Homocysteine, Metabolic syndrome

## Abstract

This review discusses the differing effects of enzyme-inducing and non-inducing antiseizure medications on cardiovascular risk and their implications for the management strategies of epilepsy patients. Traditional risk markers, including low density lipoprotein, high density lipoprotein and triglycerides, can be altered by both enzyme induction and inhibition. Other markers of vascular risk, including c-reactive protein, non-high-density lipoprotein and homocysteine, are affected by antiseizure medications, although adults and children may have different responses. The overall atherosclerotic risk picture is more complex due to indirect effects such as neuroendocrine function and the metabolic syndrome. Large scale data shows an evolving understanding of cardiovascular risk. Long term risks of enzyme inducing antiseizure medications and valproic acid are apparent when studies examine medications individually. Finally, effects of antiseizure medications on cardiac rhythm and possibly autonomic control are discussed with respect to their clinical relevance to the practicing clinician.

## Highlights

• Differentiates cardiovascular risk of enzyme-inducing antiseizure medications.
• Discusses ASM effects on less conventional markers (CRP, non-HDL, homocysteine).
• Examines age and sex-specific responses to ASMs, providing clinical insights.
• Highlights indirect ASM cardiovascular risks, including metabolic effects.

## 1 Introduction

The management of epilepsy primarily involves the use of antiseizure medications (ASMs), which aim to reduce the frequency and severity of seizures. As clinicians, we focus on protecting our patients’ brains from the cumulative effects of uncontrolled epilepsy. ASMs can significantly improve the quality of life for epilepsy patients by improving seizure-freedom, yet their use is not without risks. Among the various adverse effects associated with ASMs, cardiovascular risk has emerged as a significant concern.

Antiseizure medications confer may cardiac risk by accelerating atherosclerosis, but their effects are heterogeneous. In addition, pro-arrhythmic and autonomic effects of ASM carry additional cardiac risk. Here we will explore the differing effects of ASMs on cardiovascular risk, with particular emphasis on the differences between enzyme-inducing and non-inducing ASMs, and how these differences may influence our management strategies for epilepsy patients. A thorough understanding of which medications confer which risks, and how to monitor our patients for cardiovascular risk mitigation, is essential to save the brain without risking the heart.

## 2 Risk of atherosclerosis

ASMs are a diverse group of medications that modulate various neuronal mechanisms to control seizures. They can be broadly categorized as enzyme-inducing ASMs (EIASMs) and non-enzyme-inducing ASMs (NEIASMs). EIASMs, such as phenytoin, carbamazepine, and phenobarbital, are known to broadly induce hepatic enzymes, particularly the cytochrome P450 (CYP) enzyme system. This induction can accelerate the metabolism of not only the ASMs themselves but also other concomitantly administered drugs, leading to significant drug-drug interactions. Conversely, NEIASMs, including valproate, lamotrigine, and levetiracetam, do not significantly induce these enzymes and thus have a lower potential for such interactions (see Table 1).

**Table 1:** Categorization of ASMs as EIASMs, weak enzyme inducing ASMs, mixed effects, NEIASMs, or enzyme inhibiting NEIASMs.

Enzyme Inducing ASM (EIASM)	Weak enzyme inducing ASM	Mixed effects	Non-Enzyme Inducing ASM (NEIASM)	Enzyme Inhibiting NEIASM
Carbamazepine Phenytoin Phenobarbital Primidone	Eslicarbazepine Oxcarbazepine Rufinamide	Cannabidiol Cenobamate Clobazam Perampanel	Brivaracetam Ethosuximide Lacosamide Lamotrigine* Levetiracetam Gabapentin Tiagabine Zonisamide Topiramate**	Valproic Acid

*Induces the uridine 5′-diphospho glucuronosyltransferase (UDG) pathway.

**CYP3A induction only at high doses.

The biochemical pathways that synthesize hormones and cholesterol are modulated by the CYP system [1] and enzyme induction can increase cholesterol production by the liver [2]. It is postulated that this induction reduces the liver's feedback control over 3-hydroxy-3-methylglutaryl-coenzyme A (HMG-CoA) reductase, the rate-limiting enzyme in cholesterol production [3]. Consequently, this leads to elevated total cholesterol and low-density lipoprotein (LDL) levels, increasing vascular risk in patients taking EIASMs [2]. For this reason, atherosclerotic effects of antiseizure medications are thought to be more prevalent in the inducers of the CYP enzyme system, but a more nuanced understanding of these differences reveals that this property is not the only determinant of cardiovascular risk and that ASM risks need to be studied according to individual drug and among different patient populations.

## 3 Traditional risk markers

The most widely used markers of vascular risk are total cholesterol (TC), low density lipoprotein cholesterol (LDL), high density lipoprotein cholesterol (HDL) and triglycerides. Carbamazepine (CBZ) and valproic acid (VPA) are the most well studied EIASM and NEIASM, respectively. Investigators typically study either pediatric or adult populations, so that direct data comparing the two age cohorts is lacking, but a synthesis of the literature shows common themes regarding the effects of EIASM and NEIASM in the different populations, with more subtle distinctions with respect to the influence of sex.

### 3.1 Adults

Traditional risk marker studies were systematically reviewed [4] for ASM-treated epilepsy patients over the age of 15 years with the finding that CBZ had the strongest evidence for raising total cholesterol, HDL and LDL. VPA did raise LDL and triglycerides and lowered HDL in several studies. Overall, there were inconsistent findings and effect sizes were not calculated. LDL/HDL ratios were increased among both CBZ and VPA treated adults relative to lamotrigine treated patients or controls [5]. When switching from CBZ or phenytoin to levetiracetam or lamotrigine, adults did show rapid decreases in total cholesterol and triglycerides [6], and total cholesterol was higher in CBZ versus lamotrigine or levetiracetam-treated elderly patients [7]. Based on these findings, both EIASM and the NEIASM VPA can cause deleterious effects on traditional lipid markers since EIASM can increase most cholesterol fractions, but VPA can preferentially decrease HDL. The non-inhibiting drugs levetiracetam and lamotrigine have minimal effects on traditional risk markers of atherosclerosis in adults.

### 3.2 Children

In children, CBZ-treated groups had higher TC and LDL than controls in multiple studies [8], [9], [10], [11]. In some studies [8], [11] HDL levels were not different between CBZ and non-CBZ treated children, but in others there were higher HDL levels in CBZ treated children compared to healthy controls [9], [10]. The effect of VPA on LDL levels in children is not consistent, and it likely does not have a substantial effect overall [8], [11]. The decrease in HDL caused by VPA seems to be more apparent in girls who were post-puberty, possibly due to hormonal effects [12]. LDL was elevated amongst CBZ, VPA, topiramate and lamotrigine treated children, but not among levetiracetam treated children [13].

## 4 Other risk markers

### 4.1 CRP and hsCRP

C-reactive protein (CRP) is an acute phase reactant which is elevated in multiple medical conditions, including infection, malignancy, and autoimmune disease. Its role in cardiovascular risk assessment has expanded as chronic small elevations are associated with the development of atherosclerosis [14]. High sensitivity-CRP (hs-CRP) is a costlier test that is more sensitive to detect low levels of this [15]. CRP elevations were found in CBZ- relative to lamotrigine- or levetiracetam- treated patients in a repeated measures study on elderly adults [7]. While CBZ increases hsCRP in adults, children treated with the drug did not show the same effect [16], [17]. Underlying mechanisms for these age-related differences are not fully understood and warrant further investigation.

Chuang et al found that patients receiving VPA monotherapy had moderately increased hs-CRP levels compared to healthy controls [16]. The elevation in hs-CRP was less pronounced than that observed with some other antiepileptic drugs, such as carbamazepine. The underlying mechanism for VPA-induced hs-CRP elevation is not fully understood. Management of elevated hs-CRP in epilepsy patients can include consideration of statin therapy in patients who are already intermediate risk from a Framingham study score [15], but switching from EIASM to NEIASM did show reversal of CRP among numerous other improvements in cardiovascular risk markers and should be offered [6].

### 4.2 Non-HDL- and vLDL-cholesterol

Non-HDL cholesterol is calculated by subtracting HDL from total cholesterol and includes LDL, very-low-density lipoprotein (LDL) vLDL and other cholesterols. It can be a more powerful predictor of cardiovascular disease than LDL alone [18]. Studies on adults with EIASM compared to NEIASM suggest that non-HDL cholesterol is raised by ASM. Switching adult patients from CBZ or phenytoin to NEIASM resulted in substantial reductions in non-HDL [6]. non-HDL was elevated in adults on either CBZ or VPA [19], and also elevated in CBZ-treated compared to lamotrigine or levetiracetam-treated elderly patients [7]. Switching adult males from adjunctive CBZ to lacosamide may reduce non-HDL when patients are on levetiracetam [20]. Adult patients on EIASM may therefore benefit from a transition to a NEIASM.

Very low-density LDL is the precursor of LDL, and the main carrier of triglycerides within the circulation. In addition to triglycerides, it consists of phospholipids, cholesterol, and several apolipoproteins, of which Apolipoprotein B is its structural protein, and elevated vLDL is implicated in risk of cardiovascular disease as well as hepatic conditions. Chen et al’s examination of vLDL’s association with ASM has been focused mostly in pediatric age groups [21], with no significant difference of vLDL in children treated with CBZ versus controls [9], [22], [23]. In adults, however, both VPA and CBZ treated groups had significantly higher vLDL values than control group patients, while patients treated with lamotrigine had vLDL values that were similar to those of control group patients, indicating potential advantages in terms of lipid profile. CBZ-treated patients had vLDL values that were significantly higher than VPA-treated patients [5]. Adults appear more susceptible to ASM induced changes in vLDL levels than children, which may indicate increased risk of cardiovascular complications.

### 4.3 Homocysteine

Homocysteine is an amino acid linked to cardiovascular risk, especially stroke. Using various different control populations, CBZ-treated adults had higher homocysteine levels in several studies [24], [25], [26]. Children treated with CBZ did not have significant elevations in homocysteine compared to their healthy or matched controls [27], [28], [29]. The reason for the difference in CBZ’s effect on adults and children is not known, but this is consistent with what is seen in other markers of vascular risk, including CRP. VPA-treated adults and children had only modest or no elevations in homocysteine in those same studies [24], [25], [26], [27], [28], [29], although a meta-analysis did show that valproic was associated with increased homocysteine levels [30]. The robust effect of CBZ is likely due to increased hepatic metabolism of cofactors involved in homocysteine breakdown, including folic acid and B vitamins [31]. For VPA-related hyperhomocysteinemia, the mechanism is unknown but is likely unrelated to metabolism of cofactors [26]. Long term use of levetiracetam in children did not cause elevations in homocysteine [29], and switching from phenytoin to lamotrigine or levetiracetam reversed hyperhomocysteinemia in adults, among other changes [6], which further supports the notion that enzyme induction is responsible for CBZ-related increases in homocysteine.

## 5 Data on long-term cardiovascular risk

A series of large-scale studies have shown an evolving understanding of the long-term cardiovascular risks of EIASM. A United States claims-based database study of 150,124 patients prescribed new ASM with a minimum of 6 months follow up found no statistically significant increase in ischemic coronary or cerebrovascular events among EIASM but did find a trend toward higher hazard ratios for cerebrovascular disease in EIASM with increasing duration of therapy. Subgroup analysis of individual ASM did show increased risk of either type of ischemic event for carbamazepine compared to topiramate or gabapentin prescriptions [32]. A United Kingdom study later investigated a larger cohort with at least one prescription of ASM and found a slight increase in myocardial infarction after 24 months of therapy [33]. A study of patients between 2003 and 2017 found an increase in cardiovascular disease among epilepsy patients as a whole compared to a matched control population but did not find that there was a difference in cardiovascular disease between patients on EIASM and NEIASM; in this study, VPA was the most commonly prescribed NEIASM [34].

Analyzing a cohort of 31,479 adult participants over an extended period, Josephson et al found that epilepsy patients exposed to EIASMs had a higher incidence of cardiovascular disease. Specifically, the study highlights that individuals who received four consecutive prescriptions for EIAEDs at the time of diagnosis had a higher hazard ratio for developing cardiovascular disease compared to those who were not exposed to EIASM [35]. This study’s long follow-up period made it possible to detect significant elevations in hazard ratios for EIASM that emerged for patients after 10–25 years.

A global analysis of antiseizure medications and cardiovascular risk performed by Mayer et al included over 30,000 patients [36]. Rather than combine EIASM into one group, they compared cardiovascular disease between three individual ASMs: CBZ, lamotrigine, and VPA. Both VPA acid and CBZ had higher cardiovascular events than lamotrigine, and the incidence of death from cardiac and noncardiac causes was higher with VPA than for CBZ or lamotrigine. It is possible that earlier studies did not find elevated risks of EIASM due to grouping of NEIASM with VPA and attenuating the relative risk of EIASM.

## 6 Alternative effects on atherosclerosis

In Josephson et al’s study, less than 5 % of the hazard between EIASM use and cardiovascular disease was attributed to incident hyperlipidemia [35]. This finding implies that EIASM can affect cardiovascular risk in ways other than their effects on traditional lipid markers. Both EIASM and VPA have effects on reproductive hormones. EIASM can lower estrogen and testosterone, and VPA can cause polycystic ovarian syndrome (PCOS) in women [37]. These reproductive hormone disturbances may indirectly affect cardiovascular health. While studies that directly link cardiovascular health and ASM-related endocrine dysfunction are lacking, data on menopause shows that low estrogen can have multiple effects on cardiovascular risk markers [38]. PCOS may increase cardiac inflammation and atherosclerotic plaque formation [39].

CBZ may impact vascular risk in ways not captured by serum lipid levels. CBZ has a host of pharmacological effects, including the modulation of sodium channels and neurotransmitter release [40]. For instance, CBZ has been linked to alterations in endothelial function, which plays a crucial role in blood vessel health [41]. It induces a bioenergetics disruption to microvascular endothelial cells from the blood–brain barrier [41]. Changes in endothelial function can affect vascular tone, inflammation, and clotting mechanisms, all of which may contribute to cardiovascular health but may not be fully reflected in standard lipid assessments. Moreover, CBZ’s potential impact on vascular risk might involve interactions with other physiological processes such as oxidative stress and inflammation [42]. Understanding these effects requires further comprehensive exploration of CBZ’ actions on various physiological pathways.

## 7 Statin efficacy and EIASM

HMG-CoA reductase inhibitors (“statins”) can reduce atherosclerosis and cardiovascular risk. However, with concomitant EIASM treatment, this effect may be muted. Increased hepatic enzyme activity can lead to a faster metabolism of statins, reducing their effectiveness [7]. CBZ and statin drug interactions are common amongst epilepsy patients, and even the weaker EIASM oxcarbazepine may interact with statins [43]. Amongst elderly epilepsy patients in Finland, the most common drug-drug interaction was found to be between CBZ and statins [44]. Mintzer et al concluded that the enzyme inducing property of CBZ, rather than its sodium channel mechanism, was responsible for the elevation in lipids despite statin treatment, because the same results did not apply to lacosamide-treated patients [45]. In healthy volunteers treated with phenytoin or lamotrigine, atorvastatin area under the curve was reduced by over 50 % in patients taking both phenytoin and atorvastatin, but was unaffected for patients taking atorvastatin and lamotrigine [46]. For statin treated patients who also received either phenytoin or gabapentin, the phenytoin treated group was found to have more statin dose titrations in a large US claims database, suggesting that phenytoin reduced the effectiveness of statins [47]. There is little data on phenobarbital and primidone interaction with statins, though they would be expected to have a similar effect. The clinician should keep in mind that the risk of hyperlipidemia with EIASM is not simple to mitigate with statin therapy because of the drug interaction, and therefore switching to a NEIASM may be a better strategy to improve cardiovascular risk in patients who have both epilepsy and hyperlipidemia.

## 8 The metabolic syndrome

An important caveat to the logic that NEIASM can be less detrimental to cardiovascular health than EIASM is that VPA is associated with the metabolic syndrome, which can worsen cardiovascular health through multiple mechanisms including chronic obesity, hypertension, insulin resistance and dyslipidemia [48]. Elevated adiponectin and oxidative stress markers as well as increased insulin were found in patients treated with VPA monotherapy for one year when compared to treatment naïve epilepsy patients, but LDL and total cholesterol were decreased in these same patients [49]. A study on adult men and women found that both CBZ and VPA use were associated with the metabolic syndrome but their patterns were different between men and women [50], with women on VPA having a higher risk of metabolic syndrome than men. Also, CBZ was associated with higher HDL and valproic acid was associated with lower blood glucose. These authors postulate that low fasting blood glucose caused by valproic acid over time might lead to hunger and insulin resistance. In a group of young adult patients with epilepsy treated for at least three years with a variety of ASMs, those treated with VPA had the highest prevalence of the metabolic syndrome characterized by obesity and high triglycerides [51].

## 9 Cardiac effects of antiseizure medications other than atherosclerosis

Some EIASM and NEIASM are associated with specific arrhythmias Phenytoin is a class 1B antiarrhythmic due to its sodium channel blocking mechanism; the oral formulation of phenytoin is considered safe with regards to risk of arrhythmias [52]. Intravenous phenytoin has proarrhythmic properties due to its intravenous solute, propylene glycol, which will cause bradycardia or asystole with rapid infusion (hence the availability of a water-soluble form, fosphenytoin, which is not free from the risk of arrythmias). In patients who have a history of arrhythmias or older age, an infusion rate of 25 mg/min or less is advised, whereas most patients should have a maximum rate of 50 mg/min; While prudent, guidelines on whether patients should be on continuous cardiac monitoring during phenytoin or fosphenytoin infusions remain controversial.

Lacosamide also affects sodium channels through slow inactivation, unlike phenytoin and similar antiseizure medications which cause fast inactivation. In its oral or intravenous forms, it can cause bradycardia, AV node block, ventricular tachycardia, with overall cardiac risk of up to 1.2 % [53]. Caution using lacosamide in individuals with known cardiac disease, as well as cardiac monitoring for individuals at risk of cardiac disease while on the drug, is advised.

In 2021, the FDA issued a warning regarding lamotrigine and cardiac effects, based on in vitro data [54], which led to additional postmarketing in vitro studies that showed a weak class IB antiarrhythmic effect on human cardiac Purkinje cells. The potential effects are to shorten QT syndrome, which is the same effect as common antiarrhythmic drugs such as lidocaine. This is a theoretical problem in patients with short QT syndrome, a rare inheritable condition [55], or if taken together with other drugs that shorten QT intervals, such as antiarrhythmic medications. An international epilepsy task force recommendation did not recommend EKG for evaluation when starting lamotrigine in patients under 60 without known structural cardiac disease or risk factors [56]. A large population study in Europe did not show elevated risk of adverse cardiac outcomes such as the need for implanting a pacemaker, cardiac or all-cause mortality in users of lamotrigine compared to nonusers with similar rates of epilepsy [57], suggesting that the clinical impact of lamotrigine on cardiac risk is insignificant. These findings were further confirmed in the global network study that showed lower rates of cardiovascular risk in lamotrigine versus CBZ or VPA users [36].

There has been some speculation as to the connection between ASM and autonomic regulation. Both parasympathetic and sympathetic markers were seen to have been dysregulated on a study of epilepsy patients before and after treatment with CBZ [58]. A study exploring the effects of ASM on HRV and SUDEP found statistically significant associations between CBZ, levetiracetam, lamotrigine, and clonazepam and changes in sleep HRV markers. Only CBZ was found to have a significant negative impact on HRV. [59]. Another study found no association between HRV and ASM [60]. Perampanel, with its glutaminergic mechanism, may be cardioprotective according to a controlled study performed on adults with adjunctive perampanel after 6 months of therapy when measuring surrogate markers of vagal tone [61].

## 10 Conclusions

This study focused on the medications for which data was more available; future studies should focus on evaluating a broader range of both enzyme-inducing and non-enzyme-inducing antiepileptic drugs to better understand their distinct cardiovascular impacts. There are clear patterns of increased risk of atherosclerosis with EIASM, but also the NEIASM valproic acid, compared to other NEIASM. These patterns can be seen with both traditional lipid markers and other markers such as CRP, non-HDL, and homocysteine, though there is some variability in these effects with age and sex. Alternative mechanisms by which ASM can increase the risk of cardiovascular disease include hormonal, metabolic, proarrhythmic, and possibly autonomic nervous system effects. These effects are not all clearly related to ASM enzyme-inducing properties, but epidemiological data on cardiovascular risk is compelling and should cause clinicians to be guarded about their use of EIASMs. Although seizure control can reduce the risk of cardiac disease through development of the epileptic heart, balancing the benefit of improved seizure control with these medications is important when considering their long-term risks and benefits on cardiovascular health.

## Ethical statement

This review adheres to scientific integrity and transparency. All data were sourced from reputable studies, with no new human subject research conducted. The authors have no conflicts of interest or affiliations that could bias the findings. This work aims solely to advance knowledge and support patient-centered care in epilepsy.

## CRediT authorship contribution statement

**Aleena Abbasi:** Writing – review & editing, Writing – original draft, Resources, Project administration, Investigation, Formal analysis, Data curation. **Bassil Abbasi:** Writing – original draft, Validation, Resources, Formal analysis, Data curation. **Scott Mintzer:** Writing – review & editing, Writing – original draft, Supervision, Project administration, Methodology, Conceptualization. **Carla LoPinto-Khoury:** Writing – review & editing, Writing – original draft, Validation, Supervision, Resources, Project administration, Methodology, Investigation, Formal analysis, Data curation, Conceptualization.

## Declaration of competing interest

The authors declare that they have no known competing financial interests or personal relationships that could have appeared to influence the work reported in this paper.
